# Acoustic activity of bats at power lines correlates with relative humidity: a potential role for corona discharges

**DOI:** 10.1098/rspb.2022.2510

**Published:** 2023-03-29

**Authors:** Jérémy S. P. Froidevaux, Gareth Jones, Christian Kerbiriou, Kirsty J. Park

**Affiliations:** ^1^ Biological and Environmental Sciences, Faculty of Natural Sciences, University of Stirling, Stirling FK9 4LJ, UK; ^2^ Centre d'Ecologie et des Sciences de la Conservation (CESCO, UMR 7204), CNRS, MNHN, Sorbonne-Université, Concarneau/Paris 29900/75005, France; ^3^ School of Biological Sciences, University of Bristol, Life Sciences Building, Bristol BS8 1TQ, UK

**Keywords:** Chiroptera, corona effect, electromagnetic fields, foraging behaviour, light, noise

## Abstract

With the ever-increasing dependency on electric power, electrical grid networks are expanding worldwide. Bats exhibit a wide diversity of foraging and flight behaviours, and their sensitivity to anthropogenic stressors suggests this group is very likely to be affected by power lines in a myriad of ways. Yet the effects of power lines on bats remains unknown. Here we assessed the responses of insectivorous bats to very high voltage power lines (VHVPL; greater than 220 kV). We implemented a paired sampling design and monitored bats acoustically at 25 pairs, one pair consisting of one forest edge near to VHVPL matched with one control forest edge. Relative humidity mediates the effects of power lines on bats: we detected bat attraction to VHVPL at high relative humidity levels and avoidance of VHVPL by bats at low relative humidity levels. We argue that the former could be explained by insect attraction to the light emitted by VHVPL owing to corona discharges while the latter may be owing to the physical presence of pylons/cables at foraging height and/or because of electromagnetic fields. Our work highlights the response of bats to power lines at foraging habitats, providing new insight into the interactions between power lines and biodiversity.

## 1. Introduction

With the ever-increasing dependency on electric power in modern societies and the recent expanding focus on electrification as part of climate change mitigations [1,2], electrical grid networks are expanding worldwide. Very high-voltage power lines (VHVPL; greater than 220 kV) traverse over 300 000 km in Europe and the network is expected to grow further. In addition to collision and electrocutions [3,4], power lines may negatively affect biodiversity through various mechanisms, ranging from habitat loss and fragmentation [5,6] to the effects of electromagnetic fields (EMFs) [7,8]. By contrast, some species may benefit from the presence of power lines, possibly as a consequence of the altered environmental conditions or the management conducted under power lines [9,10]. To date, however, information on the interactions between power lines and biodiversity remains largely limited to birds [5].

Because of the wide diversity of foraging and flight behaviours exhibited by bats [11] and their sensitivity to anthropogenic stressors [12], this taxon is very likely to be affected (either negatively or positively) by power lines in a myriad of ways. Large species, species flying at height of the wires (typical height for VHVPL: approx. 10–50 m above ground, but varies with topography), and species foraging in open habitats are the most susceptible to barrier effects from VHVPL, which include mortality by collision and electrocution, and site avoidance. For instance, Tella *et al.* [13] recently documented the electrocution of 300 Indian flying foxes (*Pteropus giganteus*) in Sri Lanka while Kahnonitch *et al*. [14] revealed power line avoidance by the open-space and high-flying forager *Tadarida teniotis* in Israel. Studies assessing the effects of forest logging (e.g. clearcutting) on bats also indicate that habitat modification during the installation and maintenance of power lines could benefit open- and edge-space specialists because of increased habitat availability [15,16] but could also negatively affect clutter-adapted species that mainly forage within forest.

Furthermore, bats could be affected by less perceptible abiotic impacts of power lines such as corona discharges and EMFs. Corona discharge—an electric discharge produced by the ionization of atmospheric air surrounding the conductors—mainly occurs during wet conditions (relative humidity level greater than 80–90%) with low wind speed (less than 2 m s^−1^) [17]. It results in the production of a hissing noise (see power spectra and spectrograms of the hissing sounds in the electronic supplementary material, S1) [18] and the emission of blue and ultraviolet light over the entire conductor all along the span length (the spark generating these lights occurs at each voltage peak, i.e. *ca* 100 times s^−1^ in 50 Hz VHVPL with alternative current (AC)) and on insulators [19] (see spectral composition of corona discharge emission in air in the electronic supplementary material, S1). Noise may disrupt bat foraging behaviour and deter bats from approaching power lines either because of avoidance [20,21], noise-induced distraction [22] and/or auditory masking [23,24]. Masking may be more pronounced in species that rely on listening for prey-generated sounds to glean prey from substrates, especially if low frequency noise overlaps with the frequency hearing sensitivity of the bats. By contrast, corona discharges produce blue and ultraviolet (UV) light that can attract insects sensitive to these short wavelengths [25–27]. Insect attraction may, in turn, attract ‘light tolerant’ insectivorous bats to power lines, as is the case for streetlamps, especially those that emit short wavelength light [28]. Species that use magnetic cues are particularly affected by Earth's EMFs generated by power lines [29] and this is the case of many bat species which use EMFs for homing, roosting and foraging [30–32]. Power lines generate extremely low-frequency EMFs (50–60 Hz) but also EMFs at higher frequency (mainly between 150 kHz and 30 MHz) when corona discharges occur [33]. EMFs could exert avoidance responses in bats and disrupt foraging behaviour as documented for other mammals [34] and as also observed in bats at the much higher frequencies emitted by radar [35,36] (but see [37]).

Since bats are expected to respond either positively or negatively to power lines depending on foraging guild, the net effect of power lines on bat communities is not obvious and has not been assessed. In this study, we examined the responses of insectivorous bats to VHVPL in the field. The aim was to assess the potential effects of VHVPL on bat activity and foraging intensity while controlling for the landscape context. We tested the hypothesis that bats would avoid power lines (i.e. lower bat foraging activity at foraging habitats near power lines). Four potential non-exclusive mechanisms for avoidance are: (i) the physical presence of these structure (pylons and cables) at foraging height which may affect high-flying species and open-space foragers; (ii) exposure to EMFs that may disrupt foraging behaviour; (iii) noise caused by corona discharges, especially for passive-listening bats; and/or (iv) corona light that may deter light-sensitive species owing to high perceived predation risk (table 1). We also tested a contrasting hypothesis that light emitted by VHVPL owing to corona discharge would attract light-tolerant bats to VHVPL (table 1). More specifically, we predicted higher bat activity and foraging intensity near VHVPL for light-tolerant bat species during wet conditions (i.e. when corona discharges occur) because of insect aggregation. This is the first study we are aware of to examine the response of bats to power lines, providing new insight into the interactions between power lines and biodiversity.

**Table 1.** Summary of *a priori* hypotheses regarding the potential effects of very high voltage power lines on bats investigated in this study. (+) indicates positive association expected, and (−) negative association.

potential effects investigated	expected responses
physical presence of power lines (pylons and cables)	high-flying and open-space species (−)
electromagnetic fields	all species (−)
corona discharges: noise	passive-listening species (−)
corona discharges: light	light-tolerant species (+)
	light-sensitive species (−)

## 2. Methods

### (a) Sampling design

We applied a paired sampling design to investigate the effects of VHVPL on bat activity and foraging intensity. The study was conducted in the eastern part of France, in Doubs and Jura counties (electronic supplementary material, S1). We monitored bats between June and August (i.e. seasonal peak of bat activity) at 25 pairs of sites over two years (2017: *n* = 10; 2021: *n* = 15). Each pair consisted of one forest edge near to VHVPL (less than 10 m; hereafter referred to as ‘treatment site’) matched with one control forest edge (hereafter referred to as ‘control site’). Forest edges were adjacent to an agricultural field (pasture or meadow). We selected forest edges as our sampling sites since they are used frequently as foraging and commuting habitats for a wide range of bat species in the study area. Treatment and control sites within each pair were matched at the local scale in terms of altitude and forest composition and at larger scales in terms of landscape composition, configuration and diversity (electronic supplementary material, S2). We aimed at selecting pairs that were separated by a minimum distance of 1000 m from each other (median of minimum distances between pairs: 2793 m, range: 972–7371 m). Sites within pairs were separated by distances between 300 and 1500 m (median: 581 m). Control sites were at least greater than 200 m from any VHVPL (range: 241–981 m). Pairs were located along six aerial transmission AC power lines, including one with maximum voltage of 225 kV (*n* = 2 pairs) and five of 400 kV (*n* = 23 pairs). General information on electromagnetic field levels generated by power lines as well as sound measurements and spectral emission of corona discharges can be found in the electronic supplementary material, S1.

### (b) Acoustic analysis

We sampled bats acoustically using SM2BAT + recorders (sampling rate: 384 kHz; Wildlife Acoustics, Concord, USA; electronic supplementary material, S3). Sites within each pair were sampled simultaneously during two to three consecutive nights, from 30 min before sunset to 30 min after sunrise. We sampled between one and four pairs per night simultaneously, representing a total of 17 and 16 sampling nights in 2017 and 2021, respectively. Sampling took place during warm nights (greater than 10°C) with low wind speed (less than 10 km h^−1^) and no rain but with varying relative humidity levels, ranging from 52 to 99% (electronic supplementary material, S4). Weather conditions were retrieved from the nearest weather station (less than 10 km; https://www.meteociel.fr/) and averaged over the entire night.

As the aim of the study was to assess the effects of VHVPL on both bat activity and foraging intensity, we used bat sound recordings to calculate these response metrics. More specifically, we used the number of bat passes recorded per night as a measure of bat activity and used the bat sequence duration to get information on bat foraging intensity [38]. We defined a bat pass as one or more echolocation calls recorded during a fixed interval of 5 s [39–41]. The fixed interval allowed us to standardize the measure of bat activity among bat species. A bat sequence duration was calculated as the duration of a series of echolocation calls with interpulse intervals less than 2 s within one or several consecutive bat passes of the same species or group of species (electronic supplementary material, S5).

We automatically identified each bat pass to the lowest taxonomic level (i.e. species or species group) using the *Tadarida* toolbox [42] which provides a confidence index associated with each bat sequence identification. We then followed recommendations from Barré *et al*. [41] to account for potential automated identification errors. Thus, we used the confidence index to retain two separate datasets: (i) one dataset of bat passes with a score greater than or equal to 0.90 (i.e. with maximum error risk tolerance of 10%); and (ii) another dataset of bat passes with a score greater than or equal to 0.50 (i.e. with maximum error risk tolerance of 50%). The former threshold is conservative and minimizes the inclusion of false positives while the latter is less cautious but retains a larger quantity of data. We conducted the statistical analyses on the dataset of bat passes with a score greater than or equal to 0.50 and checked for result consistency and robustness with the other dataset [41].

We computed the community weighted mean bat sequence duration (CWMBSD)—a metric related to foraging intensity at the bat community level—as follows:

2.1 $$\;\mathrm{CWMBS}D_{j}=\frac{\sum_{i=1}^{n}a_{ij}\;({\mathrm{MBSD}}_{ij})}{\sum_{i=1}^{n}a_{ij}},$$

where *n* is the total number of species or species group recorded, *a_ij_* is the number of bat sequences of the species or species group at a given site-night combination *j*, and MBSD_i*i*_ is the mean bat sequence duration of the species or species group at a given site-night combination *j*. Beforehand, *a_ij_* and MBSD*_ii_* were scaled with minimum = 0 and maximum = 1 as these metrics are not directly comparable on their original scales between species or species group (notably because detection and abundance vary among species). Longer bat sequences (i.e. higher values of CWMBSD) would indicate that a bat is foraging while shorter bat sequences would suggest that a bat is commuting [38]. The CWMBSD provided a single metric that can inform about overall bat foraging intensity and that is not correlated with other response variables such as bat activity (electronic supplementary material, S6).

### (c) Landscape analysis

Landscape composition, configuration and diversity are key drivers of bat activity at local scales [43–47]. We therefore included landscape variables in our models to control for residual variations. Since bats respond to landscape variables at different spatial scales [43,48], we created 10 buffers of 50, 100, 250, 500, 750, 1000, 2000, 3000, 4000 and 5000 m radii around each sampling site using ArcGIS Desktop v10 (ESRI, Redlands, CA, USA). The large scales represent the mean maximum daily foraging movement of European bat species [49] whereas the small ones allow us to describe the near environment of the sampling sites. Within each buffer, we calculated the amount of deciduous forest, coniferous forest, grassland, cropland and urban area (Centre d'Expertise Scientifique Occupation des SOIs land cover data 2018, 10 m resolution), and computed the density of hedgerows and rivers (Institut Géographique National, Base de Données (BD) Haie and BD Carthage, respectively) and distance to the nearest river. We used the ‘landscapemetrics’ R-package to calculate the edge density (landscape configuration) and the Shannon diversity of habitats (landscape diversity).

### (d) Statistical analysis

We conducted a series of (generalized) linear mixed-effect models (GLMMs; ‘glmmTMB’ package) to assess the effects of VHVPL on bat activity and foraging intensity. The 11 response variables were the number of bat passes per night for species or group of species (i.e. species-specific bat activity and composite bat activity, 10 response variables), as well as the community weighted mean bat sequence duration per night (i.e. bat foraging intensity, one response variable). Composite bat activity refers to the inclusion of species-specific bat activity in a single model to investigate the overall response of bats to VHVPL. Models for bat activity were fitted with a negative binomial error distribution owing to over-dispersion and coupled with a logit link function while models for bat foraging intensity were fitted with a Gaussian distribution. We considered site identity nested within pair as random effects because bats were surveyed for several nights and to account for the paired-sampling design. Moreover, we followed recommendations from Oberpriller *et al.* [50] and added the sampling year as an additional random effect, except for CWMBSD because of model non-convergence (whether sampling year was included as random or fixed effect). Species identity was added as a random factor in models for composite bat activity to account for non-independence of observations corresponding to the same species [51].

For each response variable, we built 10 candidate models (including the null one). We considered three blocks of variables (A: experiment, i.e. VHVPL versus control (categorical variable); B: weather variables (continuous variables); and C: landscape variables (continuous variables)) that we included independently (A, B, C), in combination (A + B, A + C, B + C, A + B + C), or in interaction (between blocks A and B only, i.e. A * B, A * B + C) into the models. More specifically, weather variables (block B) comprised the mean temperature at night to account for its well-known positive effect on bat (foraging) activity and relative humidity at night given that corona discharges occur in wet conditions (relative humidity levels greater than 80–90%) with low wind speed (less than 7.2 km h^−1^) [17]. Since bat sampling took place in calm conditions (see §2b, Acoustic analysis; electronic supplementary material, S2) we did not consider wind speed as a covariate. Among the nine landscape variables computed at 10 spatial scales, only the two most informative ones at their most relevant scale were considered in block C (see the electronic supplementary material, S7 for landscape variable selection). We only restrained this selection to two landscape variables to avoid collinearity issues and model overparameterization. To test the effects of corona discharges, we included the interaction between the experiment and relative humidity into the models (interaction between blocks A and B). Thus, the full models were written as follows:

2.2 $$\begin{array}{rl} & \mathrm{composite}\;\mathrm{bat}\;\mathrm{activity}∼\;\;\mathrm{experiment}\;(\mathrm{VHVPL}\;vs\;\mathrm{control})\\ & \;\times \;\mathrm{relative}\;\;\mathrm{humidity}+\mathrm{temperature}+\mathrm{landscape}\;\mathrm{variable}\;1\\ & \;+\;\mathrm{landscape}\;\mathrm{variable}\;2+1\left|\mathrm{pairID}/\mathrm{siteID}+\;1\right|\mathrm{year}+1|\mathrm{speciesID}, \end{array}$$

$$\begin{array}{rl} & \mathrm{species}\;\mathrm{specific}\;\mathrm{bat}\;\mathrm{activity}∼\;\mathrm{experiment}\;(\mathrm{VHVPL}\;vs\;\mathrm{control})\\ & \;\times \;\mathrm{relative}\;\mathrm{humidity}+\mathrm{temperature}+\;\mathrm{landscape}\;\;\mathrm{variable}\;1\\ & \;+\;\mathrm{landscape}\;\mathrm{variable}\;2+1\left|\mathrm{pairID}/\mathrm{siteID}+\;1\right|\mathrm{year} \end{array}\;(2.3)$$

2.4 $$\begin{array}{rl} \mathrm{and}\; & \mathrm{CWMBSD}∼\;\mathrm{experiment}\;(\mathrm{VHVPL}\;vs\;\mathrm{control})\\ & \;\times \;\mathrm{relative}\;\mathrm{humidity}+\mathrm{temperature}\\ & \;+\;\mathrm{landscape}\;\mathrm{variable}\;1+\mathrm{landscape}\;\mathrm{variable}\;2\\ & \;+1|\mathrm{pairID}/\mathrm{siteID}. \end{array}$$

All continuous, explanatory variables were standardized prior to their inclusion within the full models so that the regression coefficients were comparable in magnitude. We then applied an information-theoretic approach using the Akaike information criterion corrected for small sample size (AICc) to select the most parsimonious models [52] and accounted for model uncertainty by computing model-averaged predictions and standard errors across best models (ΔAICc < 6) [53–55]. We determined statistical significance using effect size statistics and their confidence intervals (CIs) [56]. In line with Muff *et al*. [57], we considered as weak, moderate and strong evidence when the 85, 95 and 98% CIs did not overlap zero, respectively. We checked for model assumptions, assessed collinearity among predictors and spatial autocorrelation of model's residuals, and validated our models (see details in the electronic supplementary material, S8).

Finally, when the interaction between the experiment and relative humidity was significant, we tested the bat activity-relative humidity relationship at control sites, and at power line sites independently, using the ‘emmeans’ package (on the full model). From the same package, we then conducted pairwise comparison of bat activity and foraging intensity between control and power line sites at each extreme value of the relative humidity gradient sampled (i.e. at 52% and 98%) at which we expect absence and presence of corona discharges, respectively. All analyses were conducted in R v. 4.1.1 [58] and references of packages used are presented in the electronic supplementary material, S9.

## 3. Results

### (a) Bat sampling

We recorded a total of 87 940 bat passes along 50 forest edges surveyed (117 detector-nights). The most detected species (or species groups) were *Pipistrellus pipistrellus* with 68 360 bat passes (77.7% of the total bat activity), followed by *Eptesicus serotinus* (7.7%), small *Myotis* bats (4.3%, hereafter referred to as ‘*Myotis* spp.’ which includes *Myotis alcathoe*, *Myotis bechsteinii*, *Myotis brandtii*, *Myotis daubentonii*, *Myotis emarginatus*, *Myotis mystacinus* and *Myotis nattereri*), *Pipistrellus nathusii*/*kuhlii* (4.3%), *Pipistrellus pygmaeus*/*Miniopterus* *schreibersii* (1.6%), *Barbastella barbastellus* (1.6%), *Nyctalus* spp. (1.6%), *Rhinolophus hipposideros* (0.6%) and *Myotis myotis*/*blythii* (0.5%, large *Myotis* bats). We recorded less than 100 bat passes of *Rhinolophus ferrumequinum* and *Plecotus* spp. and therefore disregarded these species for the analysis.

### (b) Effects of power lines on bat activity and foraging intensity

We found evidence that eight out of the 11 response variables investigated in this study (i.e. species-specific bat activity, composite bat activity and CWMBSD, a metric related to foraging intensity at the bat community level) responded to VHVPL. For each response variable, between three and 10 models were considered as best models after model selection. The null model was, however, retained amongst best models for *Myotis* spp. activity, composite bat activity and CWMBSD (electronic supplementary material, S10).

**Figure 1.**
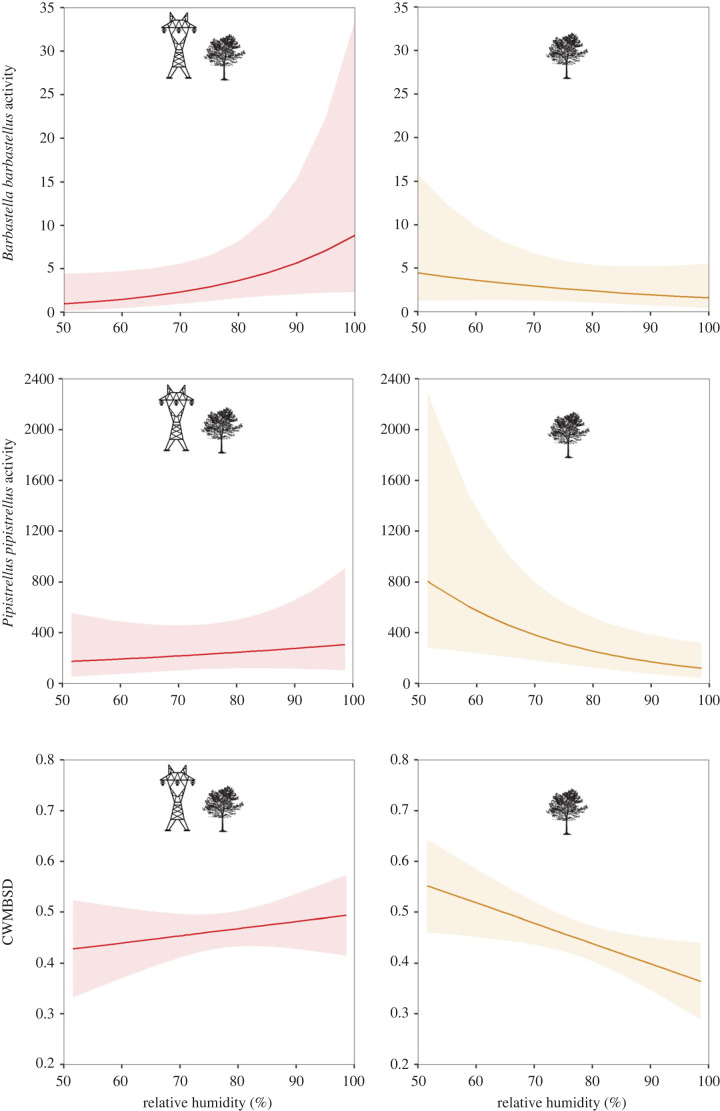
Predicted bat responses with the 95% confidence interval (CI) to relative humidity at forest edges along very high voltage power lines (≥220 kV, VHVPL) (left hand panels, red) and control sites (right hand panels, orange). Predictions were obtained from models in which the interaction between the experiment (VHVPL versus control) and relative humidity was significant with 98% CI around the estimate not overlapping zero. Activity, number of bat passes per night; CWMBSD, community weighted mean bat sequence duration (index).

**Figure 2.**
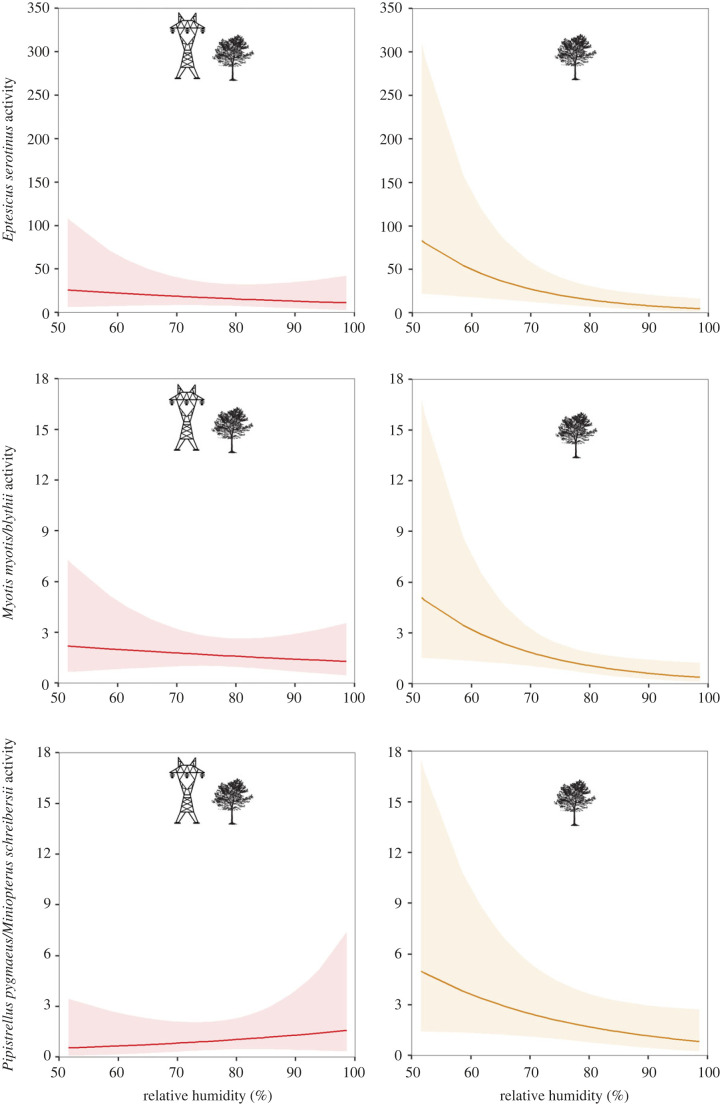
Predicted bat responses with the 95% confidence interval (CI) to relative humidity at forest edges along very high voltage power lines (≥220 kV, VHVPL) (left hand panels, red) and control sites (right hand panels, orange). Predictions were obtained from models in which the interaction between the experiment (VHVPL versus control) and relative humidity was significant with 85% CI around the estimate not overlapping zero. Activity, number of bat passes per night.

The interaction between the experiment (VHVPL versus control) and mean relative humidity at night was retained in all sets of best candidate models after model selection (electronic supplementary material, S10). Our models revealed a significant interaction (with varying strength of evidence) between mean relative humidity at night and the presence of power lines on bats for 7 out 11 of our response variables (table 2). Overall, there was lower bat activity and CWMBSD at control sites with increasing relative humidity, but at power line sites these relationships were stable or even positive. This general pattern was especially supported by our results on *B. barbastellus* and *P. pipistrellus* activity, composite bat activity and CWMBSD (figure 1; electronic supplementary material, S11 and S12) as well as on *E. serotinus*, *My. myotis*/*blythii* and *P. pygmaeus*/*Mi. schreibersii* activity, though only with weak support (table 2; figure 2; electronic supplementary material, S12).

**Table 2.** Standardized, model-averaged parameter estimates with associated standard errors (s.e.) and 85, 95% and 98% confidence intervals (CIs) of the best (G)LMMs (ΔAICc < 6) relating the effects of very high voltage power lines, humidity, and their interaction on bat taxon-specific activity, composite bat activity and community weighted mean bat sequence duration (CWMBSD). (CIs that do not overlap zero are represented in bold (85% CI: weak evidence; 95% CI: moderate evidence; 98% CI: strong evidence). A description of the most parsimonious models can be found in the electronic supplementary material, S10 and full results of the models in S12.)

variable		*Barbastella barbastellus*	*Eptesicus serotinus*	*Myotis myotis/blythii*	*Myotis* spp.	*Nyctalus* spp.	*Pipistrellus nathusii/kuhlii*	*Pipistrellus pipistrellus*	*Pipistrellus pygmaeus /Miniopterus schreibersii*	*Rhinolophus hipposideros*	composite bat activity	CWMBSD
power line versus control	est. ± s.e.	0.31 ± 0.31	−0.05 ± 0.30	0.29 ± 0.32	−0.05 ± 0.36	−0.63 ± 0.28	0.41 ± 0.35	−0.16 ± 0.30	−0.61 ± 0.46	0.39 ± 0.43	−0.25 ± 0.21	0.02 ± 0.03
	85% CI	−0.13, 0.75	−0.48, 0.39	−0.18, 0.75	−0.56, 0.46	**−1.03, −0.22**	−0.10, 0.92	−0.60, 0.28	−1.26, 0.05	−0.22, 1.01	−0.55, 0.05	−0.02, 0.05
	95% CI	−0.29, 0.91	−0.64, 0.55	−0.35, 0.92	−0.75, 0.65	**−1.18, −0.08**	−0.29, 1.10	−0.76, 0.44	−1.5, 0.29	−0.45, 1.23	−0.66, 0.16	−0.03, 0.07
	98% CI	−0.41, 1.03	−0.76, 0.66	−0.47, 1.04	−0.88, 0.78	−1.28, 0.03	−0.42, 1.24	−0.88, 0.56	−1.67, 0.46	−0.61, 1.39	−0.74, 0.24	−0.04, 0.08
relative humidity	est. ± s.e.	−0.18 ± 0.25	−0.53 ± 0.24	−0.41 ± 0.24	0.05 ± 0.15	0.32 ± 0.20	−0.41 ± 0.23	−0.38 ± 0.19	−0.35 ± 0.24	0.11 ± 0.22	−0.17 ± 0.13	−0.03 ± 0.02
	85% CI	−0.53, 0.18	**−0.88, −0.18**	**−0.76, −0.06**	−0.17, 0.28	**0.03, 0.61**	**−0.75, −0.08**	**−0.66, −0.10**	−0.69, 0.00	−0.20, 0.42	−0.36, 0.03	−0.06, 0.00
	95% CI	−0.66, 0.31	**−1.00, −0.05**	−0.89, 0.07	−0.25, 0.36	−0.08, 0.72	−0.87, 0.05	−0.76, 0.00	−0.82, 0.12	−0.32, 0.54	−0.43, 0.10	−0.07, 0.01
	98% CI	−0.75, 0.40	−1.09, 0.04	−0.98, 0.16	−0.30, 0.41	−0.16, 0.80	−0.96, 0.13	−0.83, 0.07	−0.90, 0.21	−0.40, 0.62	−0.48, 0.15	−0.08, 0.02
power line versus control : relative humidity	est. ± s.e.	0.73 ± 0.29	0.48 ± 0.29	0.49 ± 0.31	0.19 ± 0.26	−0.26 ± 0.26	0.39 ± 0.30	0.59 ± 0.22	0.68 ± 0.36	0.34 ± 0.32	0.44 ± 0.16	0.06 ± 0.02
	85% CI	**0.32, 1.14**	**0.06, 0.89**	**0.05, 0.93**	−0.19, 0.56	−0.64, 0.11	−0.03, 0.82	**0.25, 0.92**	**0.17, 1.20**	−0.12, 0.80	**0.22, 0.67**	**0.03, 0.10**
	95% CI	**0.17, 1.29**	−0.10, 1.05	−0.12, 1.09	−0.33, 0.70	−0.78, 0.25	−0.19, 0.97	**0.12, 1.05**	−0.02, 1.39	−0.29, 0.97	**0.13, 0.75**	**0.01, 0.11**
	98% CI	**0.06, 1.40**	−0.20, 1.16	−0.23, 1.21	−0.43, 0.80	−0.88, 0.35	−0.30, 1.08	**0.04, 1.13**	−0.16, 1.52	−0.41, 1.09	**0.08, 0.81**	0.00, 0.12

**Figure 3.**
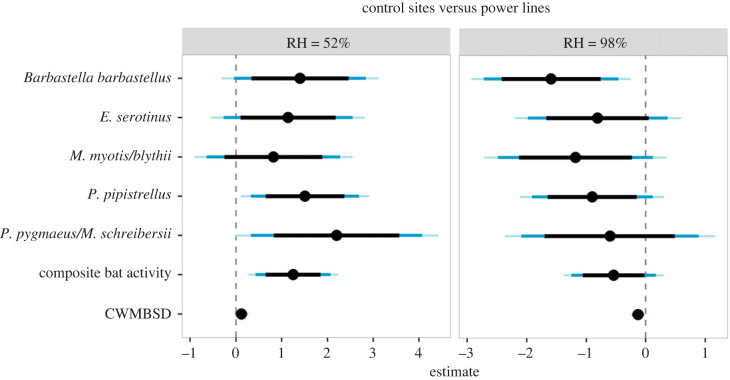
Pairwise comparisons of bat activity and foraging intensity (CWMBSD) between control sites and power lines at each extreme value of the relative humidity (RH) gradient sampled (i.e. 52 and 98%). Estimates and associated 85%, 95% and 98% confidence intervals of the comparisons are represented with black points and black, dark-blue and light-blue bars, respectively. Positive estimates indicate higher bat activity and increased foraging intensity at control sites compared to power lines.

The pairwise comparison of bat activity and CWMBSD between control sites and power lines at the extreme values of the relative humidity gradient further indicated that relative humidity mediates the effects of power lines on bats. For a low relative humidity level (here 52%) at which no corona discharges are expected we found avoidance of power lines by bats—i.e. lower bat activity and CWMBSD at power lines compared to control sites (figure 3). Conversely, for a high relative humidity level (here 98%) at which corona discharges occur, we found bat attraction to power lines with higher activity and CWMBSD at power lines compared to control sites (figure 3). This general pattern was detected for most response variables but with varying strength of evidence (figure 3).

**Figure 4.**
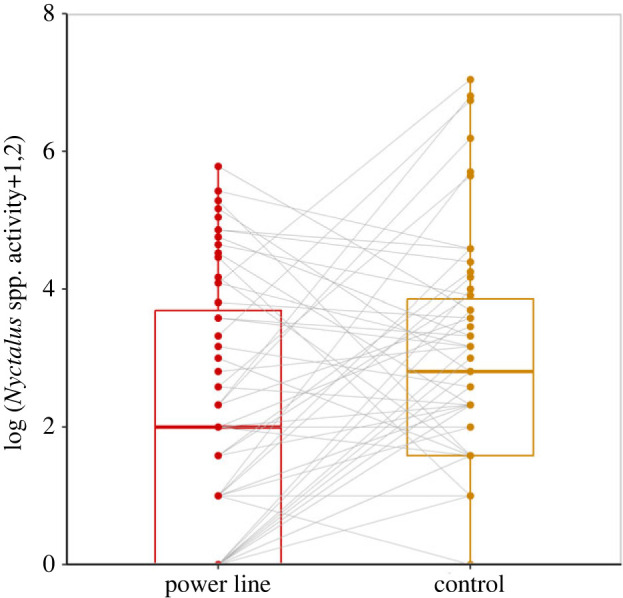
Boxplot of *Nyctalus* spp. activity (number of bat passes per night on a logarithm scale to the base 2) recorded at forest edges along very high voltage power lines (≥220 kV) and control sites. Dots represent raw data with paired sites and nights linked with a grey line. The boxplots display the interquartile range box (top line = 75% of the data ≤ this value; middle line = median; lower line = 25% of the data ≤ this value) and the lower and upper whiskers (minimum and maximum data points).

Furthermore, we found moderate evidence that *Nyctalus* spp. activity was negatively affected by the presence of very high voltage power lines (table 2; figure 4), irrespective of meteorological conditions. *Nyctalus* spp. activity was almost twofold lower (i.e. 46% reduction) at forest edges located near power lines compared to matched control sites. No evidence for an effect of power lines on *Myotis* spp., *P. nathusii*/*kuhlii* and *R. hipposideros* activity was detected (table 2).

Finally, when comparing model outputs between the two bat datasets (i.e. datasets with acoustic data identified at the 50% and 10% error risk tolerance, respectively), influential variables showed consistent patterns (electronic supplementary material, S13). We are therefore confident in our results as they are not sensitive to the rate of error risk tolerance.

### (c) Effects of landscape variables on bat activity and foraging intensity

Landscape variables were retained in all most parsimonious models (electronic supplementary material, S9) and had significant effects on species-specific bat activity but no effect on CWMBSD (a metric related to foraging intensity at the bat community level) and composite bat activity (electronic supplementary material, S13). The effect of landscape compositional heterogeneity (i.e. Shannon diversity of habitats) at broad spatial scale (≥2 km radius scale) was always positively associated with bat activity, including *E. serotinus*, *My. myotis*/*blythii, P. nathusii*/*kuhlii* and *P. pipistrellus*. The density of hedgerows was the most selected landscape variable in models on species-specific bat activity (present in six out of nine models) but had contrasting effects: the activity of *B. barbastellus*, *R. hipposideros* and *P. pygmaeus*/*Mi. schreibersii* increased with hedgerow density while the opposite was true for *My. myotis*/*blythii* and *Nyctalus* spp. and no significant effect was found for *Myotis* spp. Additionally, the amount of coniferous forest at 4 km radius scale had a negative effect on both *B. barbastellus* and *P. pipistrellus nathusii*/*kuhlii* activity whereas *R. hipposideros* was less active with increasing deciduous forest cover at the small spatial scale (50 m radius scale). The density of river at 5 km radius scale was positively related with the activity of *P. pipistrellus* and *P. pygmaeus*/*Mi. schreibersii*. Surprisingly, grassland cover had negative effects on open-space forager activity (*E. serotinus* and *Nyctalus* spp.).

## 4. Discussion

Our field study shows conclusively that bat activity and foraging intensity at foraging habitats are affected by the presence of VHVPL. Overall, our results indicate that relative humidity mediates the effects of power lines on bats as we detected bat attraction to power lines at high relative humidity levels (i.e. when corona discharges occur) and avoidance of power lines by bats at low relative humidity levels (i.e. when no corona discharges are expected). While the underlying mechanisms remain to be tested, the former result is consistent with expectations from our hypothesis that light emitted by VHVPL owing to corona discharges would attract insects and therefore increase bat foraging intensity near VHVPL. From the four potential non-exclusive mechanisms that could explain power line avoidance by bats, our results suggest that any negative effects of VHVPL on bats are most likely owing to the physical presence of the power lines and/or exposure to extremely low frequency EMFs. Noise, light and high frequency EMFs arising from corona discharges seemed to play no role in explaining avoidance of power lines by bats.

We found that relative humidity exacerbated bat activity and community-level foraging intensity at foraging habitats near power lines compared to control sites. Among many other factors, corona discharges at power lines mainly occur during wet conditions [17] and result in the emission of UV and blue light with peaks within the range of 230–440 nm [19]. The so-called ‘corona light’ has shown to be responsible for power line avoidance by reindeer [59], but given its spectrum it may attract nocturnal insects [25,26] and thus bats. Interestingly, we found attraction to power lines in both light-tolerant (e.g. *P. pipistrellus*) and light-sensitive (e.g. *B. barbastellus*) bat species and no attraction or avoidance in other light-sensitive bats present in our study area including species (e.g. *Myotis* spp.) that may perceive UV and short wavelength blue light [60,61]. While we did not specifically test the corona effect on insect prey, previous studies have highlighted clear positive relationships between insect abundance and bat (foraging) activity [62–64]. Furthermore, our results not only suggest changes in bat activity but also changes in bat behaviour with increased foraging intensity near power lines when corona discharges occur (i.e. at high relative humidity levels), thus implying that changes in bat activity mirror bat responses to their insect prey. Direct measurements of insect abundance in relation to corona discharges are however needed to confirm the process involved. Indeed, other factors such as high frequency EMFs owing to corona discharges could also be at play (e.g. by disrupting sensory orientation of bats), but so far the only studies assessing the effects of EMFs on bats have suggested either a negative effect on bat activity and foraging intensity [35,36] or no impact [37], albeit at much higher frequencies. Further research is also required to identify the distance at which this cascading effect operates to fully appreciate the mechanisms involved.

Corona discharges can also cause a hissing noise and we predicted power line avoidance by passive-listening bats—here *My. myotis*/*blythii* [65] and *Plecotus* spp. [66]—during wet conditions. We could not test this hypothesis with *Myotis* spp. in a robust way as this species group includes both active- (e.g. *My. nattereri*) and passive- (e.g. *My. bechsteinii*) listening bat species [67], even though the former is more likely to forage at forest edges than the later. However, our results on *My. myotis/blythii* refute the power line avoidance hypothesis owing to ‘noise disruption’ since its activity at forest edges near power lines was less affected by relative humidity compared to control sites. We did not have enough records of *Plecotus* spp. to conduct the analysis on its activity or occurrence and the potential effects of noise produced by VHVPL on this species group cannot be excluded for two reasons. First, *Plecotus* spp. cease echolocating during the hovering phase of gleaning attacks [68] and are therefore more likely to be disrupted by noise. Second, *Plecotus* spp. have exceptionally high hearing sensitivity with a threshold of −20 dB sound pressure level for hearing frequencies between 12 and 19 kHz [69], i.e. in the high frequency range of sound produced by the power lines.

Our prediction on power line avoidance by high-flying and open-space foragers owing to the physical structure (pylons and cables) of the power lines was supported by our results on *Nyctalus* spp. which showed significantly higher activity at control sites than near VHVPL regardless of the weather conditions. The *Nyctalus* species group includes *Nyctalus noctula* and *Nyctalus leisleri*, two open-space forager species that forage at height [70]. Our results for *Nyctalus* spp. corroborates those of Kahnonitch *et al.* [14] who revealed that the activity of the open-space and high-flying forager *T. teniotis* decreases closer to 161 KV power lines. Overhead wires greatly vary in height depending on topography (from 10 m to greater than 50 m, especially in our hilly study area) and may overlap with the flight height of *Nyctalus* spp., thus potentially representing obstacles while foraging/commuting.

However, we also observed power line avoidance by other bat species (*B. barbastellus*, *E. serotinus*, *P. pipistrellus* and *P. pygmaeus*/*Mi. schreibersii*) and reduced composite activity and foraging intensity at power lines, but only at low relative humidity levels. Wing morphology of these bat species makes them more manoeuvrable than *Nyctalus* spp. [11] and it seems unlikely that overhead wires represent physical barriers to movement for these species. As our sampling design does not disentangle the effects of extremely low frequency EMFs (50 Hz) generated by the VHVPL and the physical presence of VHVPL on bats, the potential negative effect of EMFs on bats cannot be excluded. The mechanisms underlying the avoidance of power lines by bats merits further investigation and further behavioural experiments are therefore needed to assert our findings.

To conclude, our work highlights the response of bats to power lines at foraging habitats, providing new insight into the interactions between power lines and biodiversity. We found that the effects of VHVPL on bats result from a range of potential mechanisms, with (i) corona discharges being one of the most likely factors responsible for bat attraction to power lines, and (ii) the physical presence of power lines and extremely low frequency EMFs generated by power lines the main reasons explaining power line avoidance by bats. VHVPL traverse over 300 000 km in Europe and power line avoidance by bats could result in large-scale loss, alteration and fragmentation of foraging habitat, as observed with other anthropogenic structures [71–74]. This is especially true in more arid areas where bats will not benefit from potential insect aggregation near power lines. Given that power lines can have significant conservation consequences for these protected species in Europe, these infrastructures should be considered in appropriate planning legislation and policy. We, therefore, highlight the crucial need of mitigating any negative impact that power lines may cause to bats in arid areas by applying the mitigation hierarchy with the ambition of no-net-loss, for instance by avoiding siting new power lines near important foraging habitats and offsetting habitat loss (e.g. by restoring/creating new habitats) caused by existing power lines.

## Data Availability

Data collected for this study are available in Dryad (https://doi.org/10.5061/dryad.vt4b8gtx2) [75]. Acoustic recordings are archived and available via the French citizen science programme ‘Vigie-Chiro’ (http://vigienature.mnhn.fr/page/participer-vigie-chiro), at the portal http://vigiechiro.herokuapp.com/. Data are also provided in the electronic supplementary material [76].
